# Novel semi-analytical optoelectronic modeling based on homogenization theory for realistic plasmonic polymer solar cells

**DOI:** 10.1038/s41598-021-82525-5

**Published:** 2021-02-05

**Authors:** Zahra Arefinia, Dip Prakash Samajdar

**Affiliations:** 1 Department of Photonics, Faculty of Physics, University of Tabriz, 51666-14766 Tabriz, Iran; 2 Department of Electronics and Communication Engineering, PDPM Indian Institute of Information Technology, Design and Manufacturing, Jabalpur, Madhya Pradesh 482005 India

**Keywords:** Optics and photonics, Physics

## Abstract

Numerical-based simulations of plasmonic polymer solar cells (PSCs) incorporating a disordered array of non-uniform sized plasmonic nanoparticles (NPs) impose a prohibitively long-time and complex computational demand. To surmount this limitation, we present a novel semi-analytical modeling, which dramatically reduces computational time and resource consumption and yet is acceptably accurate. For this purpose, the optical modeling of active layer-incorporated plasmonic metal NPs, which is described by a homogenization theory based on a modified Maxwell–Garnett-Mie theory, is inputted in the electrical modeling based on the coupled equations of Poisson, continuity, and drift–diffusion. Besides, our modeling considers the effects of absorption in the non-active layers, interference induced by electrodes, and scattered light escaping from the PSC. The modeling results satisfactorily reproduce a series of experimental data for photovoltaic parameters of plasmonic PSCs, demonstrating the validity of our modeling approach. According to this, we implement the semi-analytical modeling to propose a new high-efficiency plasmonic PSC based on the PM6:Y6 PSC, having the highest reported power conversion efficiency (PCE) to date. The results show that the incorporation of plasmonic NPs into PM6:Y6 active layer leads to the PCE over 18%.

## Introduction

Polymer solar cells (PSCs) have received considerable attention due to their flexible, inexpensive, and lightweight options, which motivated many researches to focus on the bulk heterojunction (BHJ) structure of polymer-fullerene or fullerene-free PSCs^1–13^. These PSCs, the active layer of which comprising of electron-donating and electron-accepting materials in a nanoscale morphology^14^, improve power conversion efficiency (PCE) by increasing the interfacial area and facilitating exciton dissociation^15,16^. However, the need to employ quite thin active layer, in the range of 50–100 nm, due to short exciton diffusion length and low carrier mobility of polymers, leads to poor light absorption^17^. To enhance light absorption in the active layer of PSCs, the incorporation of noble metal nanoparticles (NPs) is a promising method to exploit the excitation of localized surface plasmon resonance (LSPR) and light scattering for improved light harvesting and photon management, thereby increasing the PCE of PSCs^18–20^. However, the plasmonic effects in a PSC could either be advantageous or detrimental in terms of the photovoltaic performance. The limitations of plasmonic effects on the generation and transport of free electron–hole pairs in plasmonic PSCs have been addressed in Ref.^21^.

**Figure 1:**
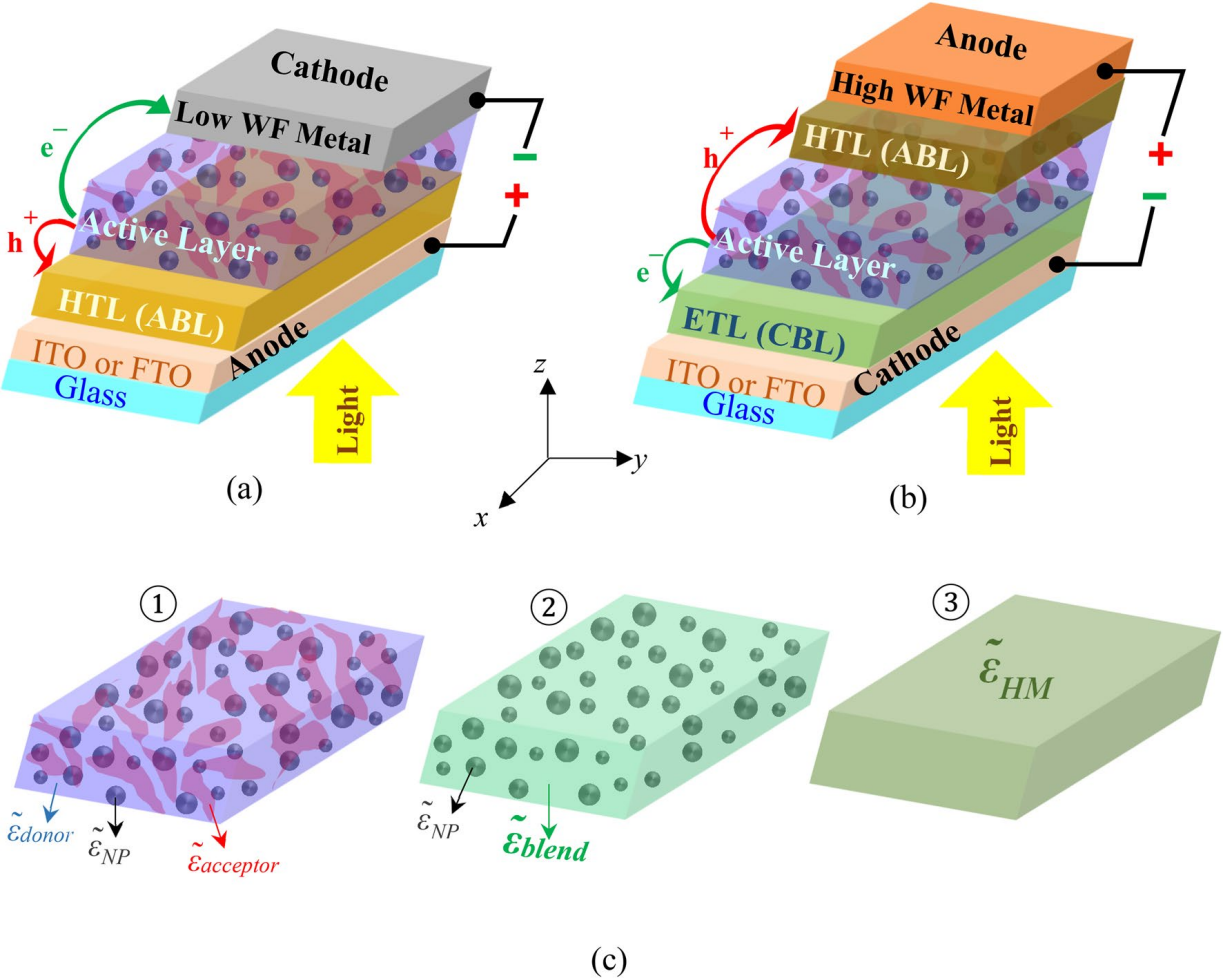
Schematic view of (**a**) conventional and (**b**) inverted structures of BHJ PSCs with the incorporation of NPs into the BHJ active layer. (**c**) Image 1 depicts the active layer of plasmonic BHJ PSC. In image 2, the blend of electron-donating material with dielectric function $\tilde{\varepsilon }_{donor}$ and electron-accepting material with dielectric function $\tilde{\varepsilon }_{acceptor}$ is replaced with a homogeneous medium with dielectric function $\tilde{\varepsilon }_{blend}$. In image 3, NPs and the surrounding homogeneous medium are replaced with another homogeneous medium with dielectric function $\tilde{\varepsilon }_{HM}$.

Incorporating NPs into different layers or at different positions of the PSCs such as into the active layer^22–35^, into the hole transporting layer (HTL) or anode buffer layer (ABL)^36–45^, into the electron transporting layer (ETL) or cathode buffer layer (CBL)^46–50^, into both the active layer and ABL^51^, and at the interface of ABL/active layer^52–55^ or anode/ABL^56,57^, with different mechanisms of enhancing device performance, has been reported in the literature. Among them, the first method, incorporating NPs into the active layer, as depicted in Fig. 1, is the subject of study of this paper. It is worth mentioning that the incorporation of NPs into the active layer of PSC, in addition to PCE enhancement, ameliorates the PSC stability by reducing structural and morphological degradation rate occurring upon prolonged solar illumination^58^.

Apart from the researches mentioned above, there are many other experimental studies on the plasmonic PSCs^59–64^, while there are a few theoretical studies for the simulation of plasmonic PSCs that almost all of them cannot be successfully applied to realistic plasmonic PSCs. To investigate the effects of certain properties of incorporating NPs, some simplifications have considered in the theoretical studies in the literature^65–73^ that should be addressed for the modeling of realistic plasmonic PSCs. Two of the simplifications include the incorporation of plasmonic NPs with the same size in an ordered array, while the experimental reports have documented the randomly blending of NPs with various sizes in the PSCs. Both of these factors cause significant changes in optical properties and consequently the PCE of plasmonic PSCs. Furthermore, the effects of interference and reflection introduced by different layers on the absorption of NPs and the effects of increased trap-assisted recombination due to NPs on the electrical properties have to be considered for realistic modeling. The implementation of these conditions to the modeling of realistic plasmonic PSCs through numerical methods leads to prohibitively long-time complex computation process due to the consideration an enormous collection of NPs, which is far beyond the reach of even modern computers. To overcome this limitation, in this study, we present a novel semi-analytical modeling for predicting the performance of the realistic structure of plasmonic PSCs to dramatically reduce computational time and resource consumptions and yet is acceptably accurate. Hence, this paper is structured as follows. The modeling is explained in Section II, where the geometrical parameters of plasmonic BHJ PSC and some assumptions we make to implement modeling are expressed in section II-A, the effect of embedded plasmonic NPs into PSCs on optical properties is described by an analytical modeling based on homogenization theory (HT) in section II-B, and the electrical properties of plasmonic PSCs are obtained in section II-C with solving the coupled equations of Poisson, continuity, and drift–diffusion. In section III, to evaluate the applicability of the semi-analytical modeling, its results are compared with the experimental results of a fabricated plasmonic PSC. Besides, the influence of the NPs parameters, including the amount of size dispersion and concentration of the NPs, on the performance of plasmonic PSCs is discussed. In section IV, based on the reported PSC with the best performance so far, a new high efficiency plasmonic PSC is proposed and investigated.

## Semi-analytical optoelectronic modeling

### Plasmonic BHJ PSC geometrical parameters and modeling assumptions

The conventional and inverted structures of plasmonic BHJ PSCs, where the NPs are incorporated into the active layer, are displayed in Fig. 1a,b, respectively. The thicknesses of active layer, ABL, CBL, anode, and cathode are *t*_*active*_, *t*_*ABL*_, *t*_*CBL*_, *t*_*anode*_, and *t*_*cathode*_, respectively, in the z direction. For the structure depicted in Fig. 1a, *t*_*CBL*_ is zero.

The illumination of both structures is through the transparent substrate and bottom electrode. The polarity of the electrodes and the current flow are reversed in inverted PSCs compared to conventional PSCs.

The morphology of the BHJ composite of active layer, the blend of donor:acceptor, can facilitate or deteriorate charge transport. For example, through the polymer in the form of fibril nanocrystal, photo-generated charges can travel efficiently from cathode to anode, while isolated polymer islands restrain charge transport and increase the recombination of charge-separated electrons and holes^74^. In this paper, the blend morphology of the BHJ composite has not been introduced in the semi-analytical modeling, and it does not account for any real interface between donor and acceptor materials. To analyze the effects of donor:acceptor interfaces, Monte Carlo (MC) or Dissipative Particle Dynamics (DPD) simulations can be implemented, which is beyond the scope of this paper^75–78^. Therefore, similar to most of the simulations of PSCs available in the literature^79–83^, it is assumed that the blend of electron-donating and electron-accepting materials with the complex dielectric functions of $\tilde{\varepsilon }_{donor}$ and $\tilde{\varepsilon }_{acceptor}$, respectively, acts as a homogeneous material with a new dielectric function of $\tilde{\varepsilon }_{blend}$ (see Fig. 1c), wavelength-dependent values of which are taken from experimental data.

Sha et al*.*^84^ have reported that the spatial location of metallic NPs in the active layer of BHJ PSCs can affect electrical properties through manipulating photo-carrier transport path. Then, with investigating three non-uniform spatial distributions of NPs (in the middle, near-anode, and near-cathode of active layer), they showed that the near-anode case resulted in the best PCE. In this paper, we consider a random distribution of NPs in the whole of active layer.

Noble metallic NPs of Ag or Au are typically incorporated into the active layer of PSCs because their absorption spectrums lie within the optical absorption band of polymer and cover nearly the entire visible and near-IR spectral region. It is assumed that the morphology of donor:acceptor blend does not alter after incorporating metal NPs, which is in good agreement with experimental reports of plasmonic PSCs with low NPs concentration^85^. Hence, first, the effect of embedded plasmonic NPs with non-uniform sizes randomly distributed in the BHJ active layer with the dielectric function of $\tilde{\varepsilon }_{blend}$ will be analytically modeled. Then, with the help of obtained results, the electrical modeling of plasmonic BHJ PSCs will be investigated.

### Optical modeling for the effect of incorporated plasmonic NPs

Optical properties of an ordered array of metal NPs incorporated into the active layer can be accurately obtained by considering a few NPs and defining their geometry exactly and then solving the Maxwell equations using numerical techniques such as finite difference time domain (FDTD)^86^, boundary element method (BEM)^87^, discrete dipole approximation (DDA)^88^, etc. In the case of irregularly incorporated NPs with various sizes, a large number of NPs must be considered for defining the geometry of active layer, and the aforementioned numerical methods are much less tractable due to the high dependence of calculation time on the size of the system. To surmount the shortcomings of numerical methods in obtaining the optical properties of a vast collection of plasmonic NPs, analytical approaches based on the HTs are methodical^89–91^. In the HTs, a complex medium formed by the inclusion of NPs into a material is replaced with a homogeneous medium that has the same optical properties as the complex medium^92–95^. Therefore, the optical properties of the active layer:NPs composite can be expressed through the HTs with the complex dielectric function of this homogeneous medium, simply, $\tilde{\varepsilon }_{HM}$.

A conventional HT is Maxwell–Garnett theory (MGT), derived from the Lorentz-Lorenz relations or the Clausius–Mossotti formula^96^. It averages over the induced electric dipole moments of individual NPs without considering the interaction between NPs to calculate the LSPR band of NPs with the same sizes^97^, so it fails to apply for NPs with size dispersion or with low interparticle distance (small distance among neighboring NPs leads to the high volume fraction of NPs). Furthermore, the MGT is based on the quasistatic limit and ignores retardation effects, so it produces significant errors for NPs with diameters that the electrostatic limit is no longer valid and the retardation effects become predominant, namely, it is restricted to the spherical NPs with the diameter (*d*) much smaller than the wavelength of incident photons (*λ*), i.e., *d* <  < *λ*^98–100^. For example, *d* < 6 nm for Au NPs and *d* < 3 nm for Ag NPs are acceptable sizes for applying the MGT^101^. Moreover, the dependence of intrinsic confinement effects, induced for very small NPs, smaller than the mean free path of conduction electrons (typically *d* < 4 nm)^102,103^, on the NPs size is not taken into account in the MGT. Indeed, the size of NPs does not explicitly appear in the MGT.

To remove the restrictions mentioned above, different extensions of the MGT have been proposed^97,104–111^. For example, a corrected version of MGT which accounts for a dipolar interaction between NPs is obtained by Markel et al.^97^, or an extension of MGT considering both intrinsic confinement and retardation effects, called Maxwell–Garnett-Mie theory (MGMT), is achieved by replacing quasistatic electric dipole polarizability with that of obtained by the Mie theory^109–111^.

In this paper, to model the optical properties of active layer:NPs composite, a modified MGMT is developed by considering the size dispersion of NPs, size-dependent intrinsic confinement for very small NPs, and retardation effects for large NPs. Therefore, it can predict the LSPR band of NPs distributed over a wide range of sizes, from very small to relatively large. Besides, light scattering within the active layer by NPs is considered by an additional contribution to the modified MGMT.

The MGMT gives the complex dielectric function of active layer:NPs composite in terms of volume fraction of NPs in the active layer (*f*), mean radius of NPs (*R̅*), and frequency- and size-dependent Mie polarizability (*α*_*Mie*_(*ω*,*R̅*)) as^90,104^:

1a $$ \tilde{\varepsilon }_{MGMT} \left( {\omega ,f,\overline{R}} \right) = \tilde{\varepsilon }_{blend} \left( \omega \right)\frac{{1 + 2\frac{f}{{\overline{R}^{3} }}\alpha_{Mie} \left( {\omega ,\overline{R}} \right)}}{{1 - \frac{f}{{\overline{R}^{3} }}\alpha_{Mie} \left( {\omega ,\overline{R}} \right)}} $$

1b $$ \alpha_{Mie} \left( {\omega ,\overline{R}} \right) = \frac{{3i\lambda^{3} }}{{16\pi^{3} \tilde{\varepsilon }^{{{3 \mathord{\left/ {\vphantom {3 2}} \right. \kern-\nulldelimiterspace} 2}}}_{blend} \left( \omega \right)}}{\text{M}}_{{^{e} }}^{(1)} \left( {\omega ,\overline{R}} \right) $$

where *λ* is the wavelength of the radiation and M_e_^(1)^(*ω*,*R̅*) is the first electric Mie coefficient expressed by:

2a $$ {\text{M}}_{{^{e} }}^{(1)} \left( {\omega ,\overline{R}} \right) = \frac{{m_{\omega } \psi_{1} \left( {m_{\omega } \overline{R}x_{\omega } } \right)\psi^{\prime}_{1} \left( {\overline{R}x_{\omega } } \right) - \psi_{1} \left( {\overline{R}x_{\omega } } \right)\psi^{\prime}_{1} \left( {m_{\omega } \overline{R}x_{\omega } } \right)}}{{m_{\omega } \psi_{1} \left( {m_{\omega } \overline{R}x_{\omega } } \right)\zeta^{\prime}_{1} \left( {\overline{R}x_{\omega } } \right) - \zeta_{1} \left( {\overline{R}x_{\omega } } \right)\psi^{\prime}_{1} \left( {m_{\omega } \overline{R}x_{\omega } } \right)}} $$

where *ψ*_*1*_ and *ζ*_*1*_ are the first order of Riccati-Bessel functions of the first and second kind, respectively, and *m*_*ω*_ and *x*_*ω*_ are defined as:

2b $$ m_{\omega } = \sqrt {\frac{{\tilde{\varepsilon }_{NP} \left( {\omega ,\overline{R}} \right)}}{{\tilde{\varepsilon }_{blend} \left( \omega \right)}}} \quad and\quad x_{\omega } = \frac{{2\pi \sqrt {\tilde{\varepsilon }_{blend} \left( \omega \right)} }}{\lambda } $$

where $\tilde{\varepsilon }_{NP}$(*ω*,*R̅*) is the size-dependent dielectric function of NPs. It should be noted that the effect of intrinsic confinement is considered in the MGMT through the implementation of $\tilde{\varepsilon }_{NP}$, instead of using the dielectric function of bulk metal ($\tilde{\varepsilon }_{bm}$), in the M_e_^(1)^(*ω*,*R̅*). By assuming that the only effect of NPs size is on the free electrons, $\tilde{\varepsilon }_{NP}$ can be derived from the Matthiessen rule by modifying $\tilde{\varepsilon }_{bm}$, described by Lorentz-Drude Model, as^112^:

3 $$ \tilde{\varepsilon }_{NP} \left( {\omega ,\overline{R}} \right) = \tilde{\varepsilon }_{bm} \left( \omega \right) - \frac{{\omega_{p}^{2} }}{{\omega \left( {\omega + i\Gamma_{0} } \right)}} + \frac{{\omega_{p}^{2} }}{{\omega \left( {\omega + i\left( {\Gamma_{0} + A_{s} \frac{{v_{f} }}{{\overline{R}}}} \right)} \right)}} $$

where *ω*_*p*_ is the plasma frequency, Г_*0*_ is the damping constant, *A*_*s*_ is the parameter depending on the scattering process of the electrons of NP surface^113^, and *v*_*f*_ is the Fermi velocity of free electrons.

The effect of size dispersion of NPs can be included in the MGMT, Eq. (1a), by considering the mean-field theory. Size dispersion leads to various electric dipole moments for NPs with different radii. Therefore, the average polarizability is calculated by weighting the polarizabilities over the relative abundance of each NP. Hence, by considering Gaussian distribution with the standard deviation of *γ* for the NPs radii, M_e_^(1)^(*ω*,*R̅*) in Eq. (1b) would be replaced with the following expression:

4 $$ \int\limits_{{R_{\min } }}^{{R_{\max } }} {\left\{ {\frac{1}{{\sqrt {2\pi \gamma^{2} } }}\exp \left[ { - \frac{{\left( {R - \overline{R}} \right)^{2} }}{{2\gamma^{2} }}} \right]} \right\}} {\text{ M}}_{{^{e} }}^{(1)} \left( {\omega ,R} \right)dR $$

where *R*_*min*_ and *R*_*max*_ stand for the smallest and largest radius of NPs in the size dispersion.

The homogenized dielectric function ($\tilde{\varepsilon }_{HM}$), describing the optical properties of the active layer:NPs composite, must address all the mechanisms resulted from inserting NPs. Therefore, in addition to the impact of plasmonic near-field due to LSPR excitation, considered through $\tilde{\varepsilon }_{MGMT}$, the effect of light scattering by the embedded NPs within the active layer must be reflected in the $\tilde{\varepsilon }_{HM}$. Because of the size dispersion of NPs, the absorption mechanism is partly attributed to enhanced LSPR near-field around the small size NPs and partly attributed to light scattering from the large size NPs^114–117^ that disperse the electromagnetic waves of the incident light. The reemitting of incident light in different directions inside the active layer leads to an increase in the optical path length^20^. The effect of enhanced optical path length by the specific angular spread of scattered light can be expressed by the Percus–Yevick correction term^89,90^. This term is added to the $\tilde{\varepsilon }_{MGMT}$, Eq. (1a), to obtain $\tilde{\varepsilon }_{HM}$ for the active layer:NPs composite as:

5 $$ \begin{aligned} \tilde{\varepsilon }_{HM} \left( {\omega ,f,\gamma ,\overline{R} ,R_{\min } ,R_{\max } } \right) & = \tilde{\varepsilon }_{MGMT} \left( {\omega ,f,\gamma ,\overline{R} ,R_{\min } ,R_{\max } } \right) \\ & \quad + i\tilde{\varepsilon }_{blend} \left( \omega \right)\frac{{16\pi^{3} \overline{R}^{3} f\left( {1 - f} \right)^{4} }}{{\lambda^{3} \left( {1 + 2f} \right)^{4} }}\left| {\frac{{\frac{{\tilde{\varepsilon }_{NP} \left( {\omega ,\overline{R} } \right) + 2\tilde{\varepsilon }_{blend} \left( \omega \right)}}{{\tilde{\varepsilon }_{NP} \left( {\omega ,\overline{R} } \right) - \tilde{\varepsilon }_{blend} \left( \omega \right)}}}}{{1 - f\frac{{\tilde{\varepsilon }_{NP} \left( {\omega ,\overline{R} } \right) + 2\tilde{\varepsilon }_{blend} \left( \omega \right)}}{{\tilde{\varepsilon }_{NP} \left( {\omega ,\overline{R} } \right) - \tilde{\varepsilon }_{blend} \left( \omega \right)}}}}} \right| \\ \end{aligned} $$

### Electrical modeling of plasmonic BHJ PSCs

The mechanism of generating electron–hole pairs and their transport in plasmonic BHJ PSCs, like pristine BHJ PSCs (without plasmonic NPs), is that the absorbed photons by the active layer cause the transition of electrons from the highest occupied molecular orbital (HOMO) of electron-donating material to its lowest unoccupied molecular orbital (LUMO) and creating neutral Frenkel excitons (FEs) with the generation rate of *G*_*F*_. Generated FEs diffuse to the donor:acceptor interface (10–20 nm) and then dissociate into electrons on the LUMO of electron-accepting material and holes on the HOMO of electron-donating material on either side of the interface with the Coulomb interaction between them, called charge transfer excitons (CTEs). CTEs will undergo recombination to FEs after a finite time unless induce to separate^118,119^. The motion of electrons causes the dissociation of CTEs into free electrons and holes moving towards the corresponding electrodes by incoherent hopping between localized states randomly distributed in space due to the field arising from the difference of the energy levels of intermediate layers or the work functions of electrodes. Therefore, for electrical modeling, following mechanisms have to be taken into account: (1) the generation, dissociation, and recombination of CTEs, (2) generation and recombination of free charges, (3) drift and diffusion of charges, and (4) the extraction of charges at the electrodes. To consider these mechanisms, several one-dimensional electrical models differing in the choice of their components, the definition of boundary conditions, and the method of solving drift–diffusion equations have been developed in the literature^80,83,120–126^. In the following, while expressing the coupled equations of continuity, drift–diffusion, and Poisson for obtaining the density of electrons and holes (*n* and *p*) and electric potential (*φ*) in the plasmonic BHJ PSCs, the effect of plasmonic NPs on the aforementioned mechanisms will be clarified.

6 $$ \frac{{\partial j_{n} }}{\partial z} = \frac{\partial }{\partial z}\left( {q\mu_{n} \left[ {n\frac{\partial \varphi }{{\partial z}} - k_{B} T\frac{\partial n}{{\partial z}}} \right]} \right) = q\left[ {G_{CT} P_{CT \to e - h} - \left( {R_{Lan} + R_{trap} } \right)\left( {1 - P_{CT \to e - h} } \right)} \right] $$

7 $$ \frac{{\partial j_{p} }}{\partial z} = \frac{\partial }{\partial z}\left( {q\mu_{p} \left[ {p\frac{\partial \varphi }{{\partial z}} - k_{B} T\frac{\partial p}{{\partial z}}} \right]} \right) = q\left[ { - G_{CT} P_{CT \to e - h} + \left( {R_{Lan} + R_{trap} } \right)\left( {1 - P_{CT \to e - h} } \right)} \right] $$

8 $$ \frac{\partial }{\partial z}\left( {\varepsilon_{HM} \frac{\partial \varphi }{{\partial z}}} \right) = q\left( {p - n + N_{A} - N_{D} - n_{RC} } \right) $$

where Eqs. (6) and (7) are the continuity equations for electrons and holes, respectively, and Eq. (8) is the Poisson equation, *j*_*n*_ and *j*_*p*_, comprised of drift and diffusion components, are the electron and hole current densities, respectively, *q* is the elementary charge, *μ*_*n*_ and *μ*_*p*_ are the mobility of electrons and holes, respectively, *N*_*A*_, *N*_*D*_, and *n*_*RC*_ are the densities of ionized acceptors, donors, and trapped charges in recombination centers, respectively, and *ε*_*HM*_ = Real[$\tilde{\varepsilon }_{HM}$ (*ω* = 0,*f*,*γ*,*R̅*,*R*_*min*_,*R*_*max*_)] is the homogenized dielectric constant of active layer:NPs composite, which shows that the plasmonic NPs affect clearly the electrical modeling of plasmonic BHJ PSCs through *ε*_*HM*_.

The right-hand side of Eqs. (6) and (7) describes generation and recombination processes, where *G*_*CT*_ is the amount of CTEs generated in the active layer which is considered as equal to the *G*_*F*_, i.e., the conversion efficiency of FEs to CTEs is considered to be unity. *G*_*F*_ is equivalent to useful absorption, i.e., the portion of incident photons of sunlight absorbed by the active layer. To calculate *G*_*F*_, the portion of parasitic photons, including absorbed photons by the non-active layers and scattered photons escaping from the PSC in all directions, is subtracted from the total number of incident photons to the plasmonic PSC. For this purpose, transfer matrix formalism that considers all optical interference effects is implemented to calculate the attenuation in each layer and the transmission and reflection at each interface layer of the plasmonic PSC, with the inputs of thickness and complex refractive index (*n͂*(*ω*) = *η*(*ω*) + *iκ*(*ω*)) of each layer^127–129^. Therefore, the effect of NPs on the *G*_*CT*_ is through the complex refractive index of the active layer:NPs composite, *n͂*_*HM*_, defined as:

9 $$ \tilde{n}_{HM} = \sqrt {\frac{{\left| {\tilde{\varepsilon }_{HM} } \right|{\text{ + Real}}\left( {\tilde{\varepsilon }_{HM} } \right)}}{2}} + i\sqrt {\frac{{\left| {\tilde{\varepsilon }_{HM} } \right| - {\text{Real}}\left( {\tilde{\varepsilon }_{HM} } \right)}}{2}} $$

It is noted that the portion of absorbed photon by NPs does not contribute to creating FEs and, therefore, is parasitic, but it is not taken into account in the semi-analytical optoelectronic modeling because Morawiec et al*.*^130^ have shown that it is insignificant in the visible part of the AM1.5G spectrum.

*P*_*CT→e–h*_ in Eqs. (6) and (7) is the probability of dissociation from CTEs to free electrons and holes defined by:

10a $$ P_{CT \to e - h} = \frac{{k_{D} }}{{k_{D} + k_{F} }} $$

where *k*_*F*_ is the rate constant of the decaying of CTEs to FEs, and *k*_*D*_ is the rate constant of CTEs separation to free electrons and holes. The analytical expression of *k*_*D*_ reported by Mihailetchi et al.^131^ is implemented in our optoelectronic modeling, which is defined as:

10b $$ k_{D} = \frac{{3q\left( {\mu_{n} + \mu_{p} } \right)}}{{4\pi a^{3} \varepsilon_{HM} }}\exp \left( {\frac{{ - E_{b} }}{{k_{B} T}}} \right)\frac{{J_{1} \left( {\sqrt {\frac{{ - q^{3} \nabla \varphi }}{{\pi \varepsilon_{HM} k_{B}^{2} T^{2} }}} } \right)}}{{\sqrt {\frac{{ - q^{3} \nabla \varphi }}{{4\pi \varepsilon_{HM} k_{B}^{2} T^{2} }}} }} $$

where *a* and *E*_*b*_ are the separation distance and binding energy of bound electron–hole pairs; respectively, *k*_*B*_ is the Boltzmann constant, *T* is temperature, *J*_*1*_ is the first order of Bessel function, and ∇*φ* is the electric field strength in the active layer:NPs composite. As seen in Eq. (10b), the impact of embedded NPs on *k*_*D*_ and consequently on *P*_*CT→e–h*_ is through the homogenized dielectric constant and electric potential of the active layer:NPs composite.

The second term on the right-hand side of Eqs. (6) and (7) describes the recombination process where *R*_*Lan*_ and *R*_*trap*_ stand for the Langevin bimolecular and trap-assisted monomolecular recombination, respectively^125,132^. The recombination of two free opposite charges created from different CTEs refers to Langevin bimolecular, and the recombination of a free charge with an immobilized charge at a trap state refers to monomolecular, where the first is defined as^133^:

11 $$ R_{Lan} = \xi C_{Lan} \left( {np - n_{1} p_{1} } \right) = \xi \left( {\frac{{q\left( {\mu_{n} + \mu_{p} } \right)}}{{\varepsilon_{HM} }}} \right)\left( {np - n_{1} p_{1} } \right) $$

where *C*_*Lan*_ = *q*(*μ*_*n*_ + *μ*_*p*_)/*ε*_*HM*_, stands for the recombination coefficient predicted by Langevin model, *ξ* is an additional reduced factor taking into account experimentally derived reduced Langevin factor^134–137^, and *n*_*1*_ and *p*_*1*_ are the characteristic electron and hole concentrations, respectively, the product of which is equal to the square of intrinsic carrier density, i.e., *n*_*1*_* p*_*1*_ = *n*_*i*_^*2*^^138^.

Trap-assisted monomolecular recombination is described by a modification of Shockley–Read–Hall rate equation (*r*_*SRH*_ (*E*))^139^ in which Gaussian density of state is considered for recombination centers (DOS_RC_ (*E*)), as follows^140^:

12 $$ R_{trap} = \int\limits_{ - \infty }^{ + \infty } {\left\{ {\frac{{N_{RC} }}{{\delta_{RC} \sqrt {2\pi } }}e^{{{\raise0.7ex\hbox{${ - \left( {E - E_{RC} } \right)^{2} }$} \!\mathord{\left/ {\vphantom {{ - \left( {E - E_{RC} } \right)^{2} } {2\sigma_{RC}^{2} }}}\right.\kern-\nulldelimiterspace} \!\lower0.7ex\hbox{${2\sigma_{RC}^{2} }$}}}} } \right\}} \left\{ {\frac{{N_{RC}^{ - 1} \left( {np - n_{1}^{{}} p_{1} } \right)}}{{\tau_{p} \left( {n + n_{1} } \right) + \tau_{n} \left( {p + p_{1} } \right)}}} \right\}dE \equiv C_{trap} \left( {np - n_{1} p_{1} } \right) $$

where the first curly bracket in the integral refers to DOS_RC_ (*E*), *E*_*RC*_ is the center of Gaussian distribution of recombination centers considered in the middle of the band gap, *δ*_*RC*_ is the width of Gaussian distribution, the second curly bracket in the integral refers to *r*_*SRH*_ (*E*), *N*_*RC*_ is the total density of recombination centers including defects, impurities, and NPs, *τ*_*n*_ (*τ*_*p*_) is electron (hole) lifetime, and *C*_*trap*_ stands for trap-assisted recombination coefficient. As reported by Wu et al.^132^, incorporating NPs into the active layer causes the increase in the recombination centers at the interfacial region of donor:acceptor. Therefore, in addition to defects and impurities density of donor:acceptor blend, the density of NPs is included in *N*_*RC*_^89^. Consequently, embedded NPs also affect *τ*_*n*_ and *τ*_*p*_ because these are inversely proportional to *N*_*RC*_^141^.

It is to be noted that the incorporation of NPs in the PSCs changes the number of photo-generated carriers. As a result, both recombination processes in plasmonic PSCs differ from NPs-free counterpart PSCs. In addition to the number of photo-generated carriers, Eqs. (11) and (12) show that *R*_*Lan*_ and *R*_*trap*_ are respectively influenced by embedded NPs through *ε*_*HM*_ and *N*_*RC*_.

Embedded NPs also influence on the Poisson equation, Eq. (8), through trapped charges in recombination centers, *n*_*RC*_, calculated as:

13 $$ n_{RC} = \int\limits_{ - \infty }^{ + \infty } {{\text{DOS}}_{RC} \left( E \right)} \left\{ {\frac{{\tau_{p} n - \tau_{n} p_{1} }}{{\tau_{p} \left( {n + n_{1} } \right) + \tau_{n} \left( {p + p_{1} } \right)}}} \right\}dE $$

where the curly bracket in the integral refers to the possibility of a recombination center being occupied by one electron.

To obtain a unique solution to Eqs. (6) to (8), it is necessary to specify appropriate boundary conditions^126,142^. They are defined for *n*(*z*), *p*(*z*), and *φ*(*z*), by assuming that the contact of active layer with ABL is hole ohmic and with CBL or cathode is electron ohmic, at *z* = 0 and *z* = *t*_*active*_ as:

14a $$ \varphi \left( 0 \right) = V - \frac{{E_{gap} - WF_{A} }}{q}; \, \varphi \left( {t_{active} } \right) = - \frac{{WF_{C} }}{q} $$

14b $$ n\left( 0 \right) = N_{c} \exp \left( { - \frac{{E_{gap} - WF_{A} }}{{k_{B} T}}} \right); \, n\left( {t_{active} } \right) = N_{c} \exp \left( { - \frac{{WF_{C} }}{{k_{B} T}}} \right) $$

14c $$ p\left( 0 \right) = N_{v} \exp \left( { - \frac{{WF_{A} }}{{k_{B} T}}} \right); \, p\left( {t_{active} } \right) = N_{v} \exp \left( { - \frac{{E_{gap} - WF_{C} }}{{k_{B} T}}} \right) $$

where *V* is the applied bias, *WF*_*C*_ and *WF*_*A*_ are the work functions of cathode and anode, respectively, *E*_*gap*_ is the effective energy band gap of active layer defined by the difference of the HOMO energy level of the donor polymer (*E*_*HOMO-do*_) and the LUMO energy level of the acceptor molecule (*E*_*LUMO-ac*_), and *N*_*c(v)*_ is the effective density of states for electrons (holes).

## Evaluation of the validity of semi-analytical optoelectronic modeling

### Choosing a fabricated plasmonic BHJ PSCs as a test case

Plasmonic BHJ PSCs based on blending of poly-(3-hexylthiphene) and phenyl-C_61_-butyric acid methyl ester, P3HT:PCBM, with the incorporation of metallic NPs with different volume fractions, sizes, and shapes have been fabricated and extensively studied^20,40,147,148^.

In the following, we will focus on the conventional structure of P3HT:PCBM PSC, reported by Stratakis and Kymakis groups^22,28,149^, and P3HT:PCBM:NPs PSC with following geometrical parameters and materials: the weight ratio of P3HT:PCBM is 1:1, *t*_*active*_ = 100 nm, the ABL is poly(3,4-ethylenedioxythiophene):poly(styrene sulfonate) (PEDOT:PSS), *t*_*ABL*_ = 40 nm, top electrode, acting as metal cathode with low WF, is aluminium, *t*_*cathode*_ = 100 nm, bottom transparent anode is ITO, and *t*_*anode*_ = 110 nm.

### Comparison to experimental data and discussion of modeling results

**Figure 2:**
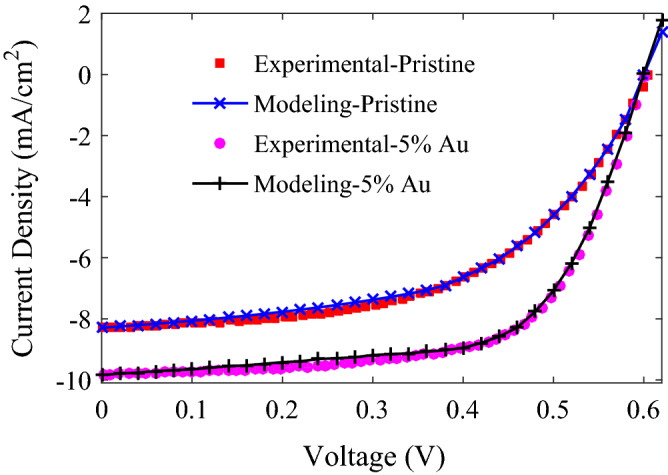
The results of the semi-analytical optoelectronic modeling for the P3HT:PCBM PSCs without embedding NPs (Pristine) and with the embedding of 5% Au NPs in the active layer under AM1.5G illumination are compared with the experimental data reported by Paci et al.^22^.

The validation of our semi-analytical optoelectronic modeling is investigated by Fig. 2. At the first stage, the pristine PSC, the ITO/PEDOT:PSS/P3HT:PCBM/Al structure without metal NPs, is simulated with the parameters indicated in Table 1. As can be found in the experimental literature, the aspects of the fabrication process of PSCs lead to a range of possible values for a parameter. From these ranges, the values of parameters in Table 1 are chosen from literature by comparing the experimental *J–V* curve with the modeling results to reach the best fitting. At the second stage, the experimental data for *J–V* characteristics of P3HT:PCBM:NPs PSC with 5% Au NPs concentration^22^, reported by Paci et al.^22^ and shown with pink solid circles in Fig. 2, are compared with the simulated *J–V* characteristics obtained by our semi-analytical modeling. Since ref.^22^ has reported that the NPs diameters with an average of 10 nm are distributed in the range of 1.5 to 20 nm, *R̅* = 5 nm, *R*_*min*_ = 0.75 nm, *R*_*max*_ = 10 nm, and *γ* = 5 nm are considered for the optical modeling of P3HT:PCBM:NPs layer. The good agreement of simulated photovoltaic parameters of P3HT:PCBM:NPs PSC with the experimental ones shown in Table 2 confirms the validity of the semi-analytical optoelectronic modeling to examine the performance of plasmonic PSCs.

**Table 1:** List of parameters used in the semi-analytical modeling.

Description	Parameter	Value	References
LUMO energy level of P3HT	*E*_*LUMO-do*_	− 3.2 eV	^143^
HOMO energy level of P3HT	*E*_*HOMO-do*_	− 5.1 eV	^143^
LUMO energy level of PCBM	*E*_*LUMO-ac*_	− 4.3 eV	^143^
HOMO energy level of PCBM	*E*_*HOMO-ac*_	− 6.1 eV	^143^
WF of Al cathode	WF_C_	− 4.3 eV	^143,144^
WF of transparent ITO anode	WF_A_	− 4.7 eV	^143,144^
Reduced Langevin factor	*ξ*	6 × 10^–4^	^145^
Electron mobility	*μ*_*n*_	3 × 10^–3^ cm^2^V^−1^ s^−1^	^146^
Hole mobility	*μ*_*p*_	1 × 10^–4^ cm^2^V^−1^ s^−1^	^146^
Electron lifetime	*τ*_*n*_	3 × 10^–8^ s	^140^
Hole lifetime	*τ*_*p*_	3 × 10^–8^ s	^140^

**Table 2:** Experimental and the semi-analytical optoelectronic modeling results of photovoltaic parameters for the P3HT:PCBM PSCs without NPs and with different concentrations of Au NPs into P3HT:PCBM active layer.

	*J*_*sc*_ (mA/cm^2^)	*V*_*oc*_ (V)	FF (%)	PCE (%)
Experimental-Pristine^22,149^	8.3 ± 0.12	0.6	53.83 ± 0.30	2.66 ± 0.05
Modeling-pristine	8.29	0.6	53.62	2.67
Experimental-5% Au^22,149^	9.77 ± 0.24	0.6	63.38 ± 0.54	3.71 ± 0.12
Modeling-5% Au	9.84	0.6	64.52	3.81
Experimental-4% Au^149^	8.90 ± 0.20	0.6	61.03 ± 0.40	3.26 ± 0.09
Modeling-4% Au	8.95	0.6	60.99	3.27
Experimental-6% Au^149^	9.60 ± 0.15	0.6	61.32 ± 0.48	3.53 ± 0.08
Modeling-6% Au	10.65	0.6	65.20	4.18
Modeling-7% Au	11.17	0.6	65.14	4.37

**Figure 3:**
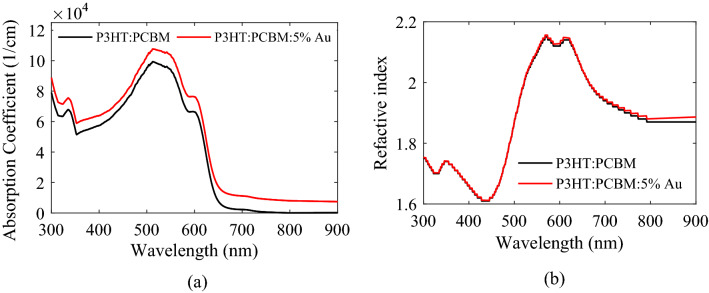
(**a**) Absorption coefficient and (**b**) refractive index of P3HT:PCBM:NPs with *f* = 0.05 (calculated by the HT) and P3HT:PCBM (experimental data^79^).

**Figure 4:**
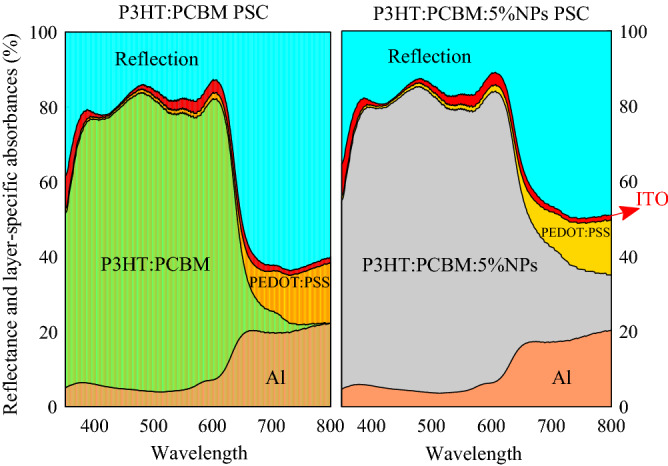
The percentage of absorbed photons in each layer and reflected photons from the P3HT:PCBM PSC (left) and P3HT:PCBM:NPs PSC (right).

It can be found from Fig. 2 and Table 2 that incorporating Au NPs into the active layer induces an improvement of PCE by 39%, which is the result of increase in the *J*_*sc*_ and FF, while the *V*_*oc*_ remains unchanged. To understand the origin of improved *J*_*sc*_, the absorption coefficient of the composite active layer of P3HT:PCBM:NPs, calculated by the HT, is shown in Fig. 3a. For comparison, the figure also shows the absorption coefficient of P3HT:PCBM. It can be seen that incorporating NPs improves the optical absorption of the active layer, resulting from the excitation of LSPR modes and multiple light scattering by the NPs simultaneously. Besides, the wide range of NPs diameter (from 1.5 to 20 nm) leads to broadband optical absorption enhancement, originating from the red shift of resonance peak of LSPR for large size NPs in size dispersion due to retardation effect and the blue shift of resonance peak for small size NPs due to intrinsic confinement effect. Another reason for improving *J*_*sc*_ is the increase in the absorbed photons percentage in the active layer (useful absorption) of plasmonic PSC. To illustrate it, the percentage of absorbed photons in each layer and the percentage of reflection, including the interference induced by electrodes and scattered light escaping from the PSC, for the P3HT:PCBM and P3HT:PCBM:NPs PSCs, calculated by transfer matrix method, are shown in Fig. 4. It is found that after incorporating Au NPs in the active layer, the percentage of reflection decreases, and the percentage of absorbed photon in the active layer increases for wavelengths above 650 nm. It should be noted that the incorporation of Au NPs also affects recombination processes. The presence of Au NPs in the active layer leads to increased trap density and consequently higher trap-assisted recombination, and strong local field around NPs results in a higher density of photo-generated excitons, *G*_*F*_, thereby leading to increased bimolecular recombination, which both recombination processes deplete the photo-generated carriers. However, the increase in the photo-generated carriers stemming from the combined effects of enhanced absorption coefficient and increased useful absorption compensates carrier loss due to the recombination processes, leading to increased *J*_*sc*_ for the P3HT:PCBM:NPs PSC.

The reason for unchanged *V*_*oc*_ can be explained with the analytic and approximate equation for *V*_*oc*_ expressed in refs.^150,151^ as follows:

15 $$ V_{oc} = \frac{{E_{gap} }}{q} - \frac{{k_{B} T}}{q}\ln \left( {\frac{{\left( {1 - P_{CT \to e - h} } \right)\left( {C_{Lan} + C_{trap} } \right)N_{c} N_{v} }}{{P_{CT \to e - h} G_{CT} }}} \right) $$

In the P3HT:PCBM:NPs PSC, the *V*_*oc*_ increase caused by the increase in the *G*_*F*_ due to the LSPR effect of embedded NPs are counteracted with the *V*_*oc*_ decrease caused by the increase in the *C*_*trap*_ due to the addition of embedded NPs to the recombination centers.

FF is pronouncedly influenced by the electrical resistivity of active layer and electrodes^152^. The inverse of electrical resistivity, electrical conductivity (*σ*), can be calculated by the real part of complex refractive index (*η*) and absorption coefficient (*α*) as follows^153^:

16 $$ \sigma \left( \omega \right) = c_{0} \varepsilon_{0} \eta \left( \omega \right)\alpha \left( \omega \right) $$

where *c*_*0*_ is the velocity of light in vacuum and *ε*_*0*_ is the vacuum permittivity.

The refractive index of the composite active layer of P3HT:PCBM:NPs calculated by the HT (*η*_*HM*_(*ω*)) is shown in Fig. 3b. It is found from Eq. (16) and Fig. 3a,b that the conductivity of P3HT:PCBM:NPs is more than the P3HT:PCBM. Increasing the conductivity is beneficial to carrier transport, leading to the increased FF of the P3HT:PCBM:NPs PSC.

**Figure 5:**
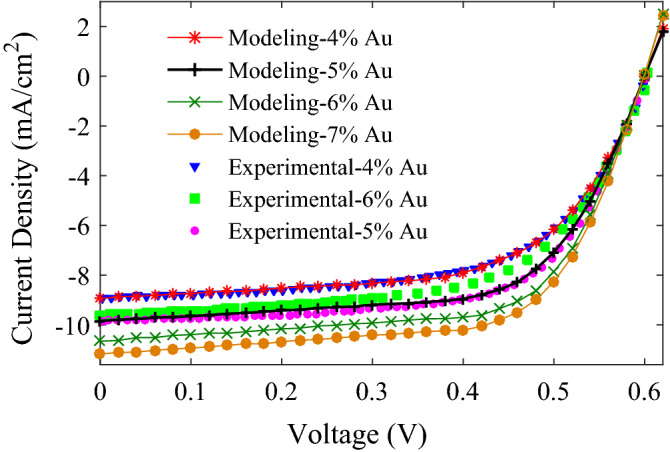
The modeling results of *J–V* characteristics for the P3HT:PCBM:NPs PSCs with different volume fractions of Au NPs (*f* changes from 0.04 to 0.07). For comparison, the experimental data reported by Spyropoulos et al.^149^ are also shown.

The influence of the concentration (volume fraction) of Au NPs on the *J–V* characteristics of the P3HT:PCBM:NPs PSCs is simulated and depicted in Fig. 5, and the calculated photovoltaic parameters are listed in Table 2. Low volume fractions of NPs, varying from 0.04 to 0.07, are considered, because incorporating NPs with high volume fraction may deteriorate the morphology of donor:acceptor blend of active layer, and may create a short circuit leading to the degradation of the electrical properties of P3HT:PCBM:NPs PSCs.

**Figure 6:**
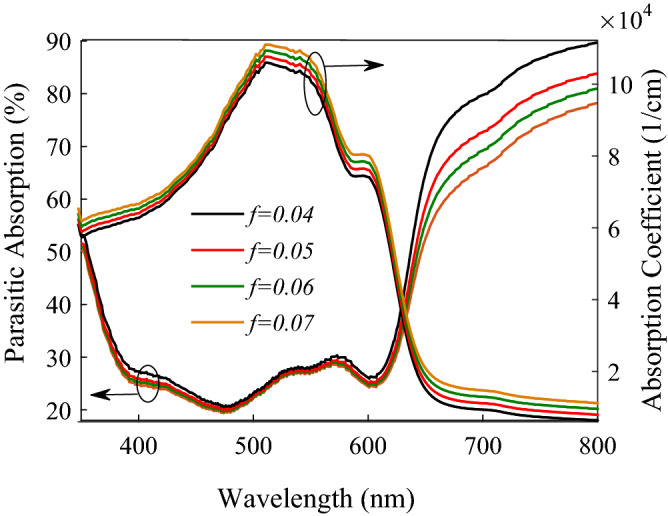
Percentage of parasitic absorption in the P3HT:PCBM:NPs PSCs (left axis) and calculated absorption coefficient of P3HT:PCBM:NPs layer (right axis) by the HT for different values of NPs concentration (*f*).

With increasing the volume fraction (*f*) of NPs in the P3HT:PCBM:NPs active layer, no variation in *V*_*oc*_ is observed from Fig. 5, but *J*_*sc*_ increases. On the one hand, increasing *f* typically increases the number of NPs in the active layer and, consequently, increases recombination centers and, as a result, decreases the number of photo-generated carriers. On the other hand, increasing *f* improves the effective absorption coefficient of the active layer, as shown in Fig. 6 (right axis), and decreases the percentage of parasitic absorption (the sum of reflection and non-active layers absorption), as shown in Fig. 6 (left axis), leading to an increase in the number of photo-generated carriers. Photo-generated carrier enhancement due to the last two reasons outweighs their loss through recombination, which leads to an increase in *J*_*sc*_ with increasing *f*.

Figure 5 and Table 2 also show the experimental data of *J–V* characteristics and respective photovoltaic parameters obtained by Spyropoulos et al.^149^. It is seen that for the P3HT:PCBM:4%NPs and P3HT:PCBM:5%NPs PSCs, there is good agreement between calculated and experimental data, but not for the P3HT:PCBM:6%NPs PSC. The poor performance of P3HT:PCBM:6%NPs PSC reported by Spyropoulos et al.^149^ is due to poor properties of NPs dispersion into the active layer resulting in NPs aggregation, affecting the plasmonic effects.

**Figure 7:**
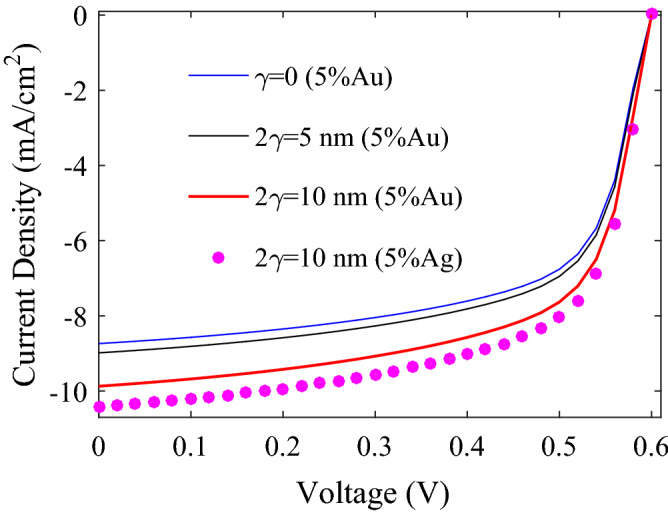
The modeling results of *J–V* characteristics for the P3HT:PCBM:NPs PSCs with 5% Au NPs and different radii dispersions (the curves of solid line) and for 5% Ag NPs (the pink solid circles).

**Figure 8:**
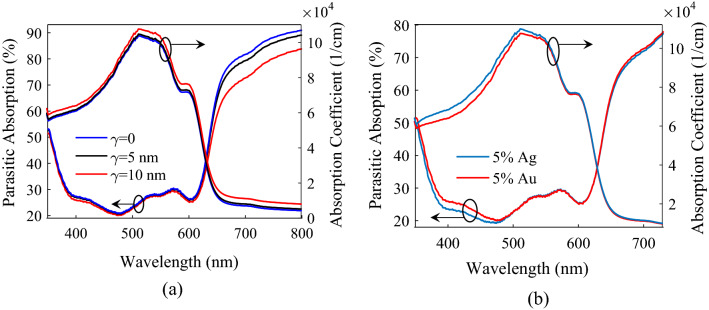
Percentage of parasitic absorption (left axis) in the P3HT:PCBM:NPs PSCs and absorption coefficient of the active layer (right axis) versus wavelength of incident light for (**a**) 5% Au NPs and different radii dispersions and (**b**) 5% Au and Ag NPs.

To investigate the effect of size dispersion on the performance of plasmonic PSCs, the *J–V* characteristics of P3HT:PCBM:5%NPs PSCs for Au NPs of 10 nm in mean diameter (2*R̅* = 10 nm) and various radius dispersions (*γ*) are shown in Fig. 7. The blue, black, and red solid *J–V* curves are respectively for the NPs with equal diameters of 10 nm (*γ* = 0), diameters in the 5 to 15 nm range (2*γ* = 5 nm), and in the 1.5–20 nm range (2*γ* = 10 nm). With increasing *γ*, *V*_*oc*_ is almost constant, and FF slightly decreases from 64.57 to 64.52%. An improvement in the *J*_*sc*_ and PCE by 2.8% and 2.6%, respectively, for 2*γ* = 5 nm and by 12.9% and 12.7%, respectively, for 2*γ* = 10 nm compared to the *γ* = 0 is achieved. *J*_*sc*_ enhancement is due to the increase in the absorption coefficient of the active layer, calculated by HT and shown in the right axis of Fig. 8a, and the decrease in the percentage of parasitic photons, shown in the left axis of Fig. 8a.

Figure 7 also shows the *J–V* characteristics of P3HT:PCBM:5%Ag NPs PSC (the pink solid circles). The parameters of incorporated Ag NPs are chosen to be the same as incorporated Au NPs reported in refs.^22,149^ (*f* = 0.05, 2*γ* = 10 nm, 2*R̅* = 10 nm, 2*R*_*min*_ = 1.5 nm, and 2*R*_*max*_ = 20 nm). The P3HT:PCBM PSC incorporated with Ag NPs exhibits higher PCE of 4.03% compared to the P3HT:PCBM:Au NPs PSC, which is 3.81%. PCE enhancement is due to improved *J*_*sc*_, from 9.87 mA/cm^2^ for Au NPs to 10.41 mA/cm^2^ for Ag NPs, implying that the improved photocurrent results from enhanced absorption coefficient of the active layer in the wavelength range of 350–550 nm and the decrease in the percentage of parasitic photons below 480 nm for the P3HT:PCBM:Ag NPs PSC compared to the P3HT:PCBM:Au NPs PSC (see Fig. 8 (b)).

## Proposing a high efficiency plasmonic BHJ PSC

As can be found from the modeling results and the experimental data of previous section, the efficiency of the plasmonic P3HT:PCBM PSCs is very low which is due to the much poorer performance of the reference P3HT:PCBM PSC, mainly resulted from intrinsic shortcomings of fullerene PCBM acceptor. The best reported PCE of single-junction PSCs with fullerene derivative acceptors, certified by National Renewable Energy Laboratory (NREL), is 11.5%^154^. Therefore, the designing of high-performance PSCs based on non-fullerene acceptors has attracted tremendous efforts in the recent years^155–165^. These efforts along with developing polymer, optimizing several aspects of BHJ morphology, and interface engineering have not only promoted the PCE of the non-fullerene PSCs to a high level of 17.23% (NREL-certified value 16.77%), but have also improved stability compared to fullerene PSCs^165^.

**Figure 9:**
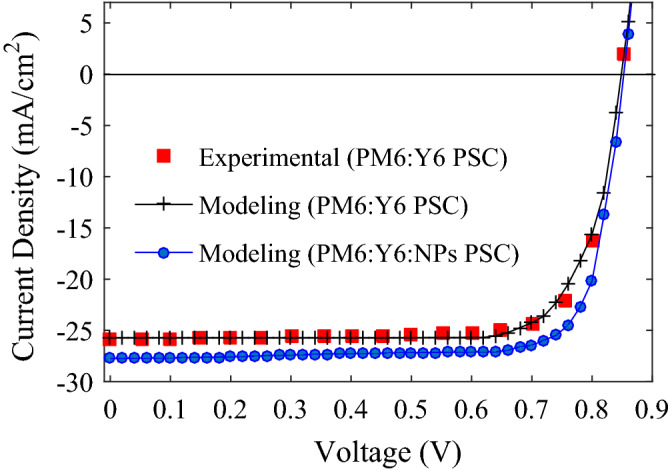
Calibrating the *J–V* characteristics of the PM6:Y6 PSC calculated by the modeling with the experimental data, reported by Li group^165^. The figure also shows the modeling results of *J–V* characteristics for the PM6:Y6:NPs PSC.

The BHJ PSC with the highest efficiency so far, reported by Li group^165^, is a conventional structure with the layers of ITO/PEDOT:PSS/PM6:Y6/PDINN/Ag, where conjugated polymer PM6 and Y6 molecule of PM6:Y6 blend are applied as p-type donor and acceptor, respectively, and aliphatic amine-functionalized perylene-diimide (PDINN)/Ag as a bilayer cathode. Based on experimental reports in the literature and our modeling results in the previous section, we foresee that, by means of incorporating plasmonic NPs into PM6:Y6 active layer, it is possible to achieve even higher efficiency than 17.23%. Therefore, our semi-analytical modeling, providing a realistic prediction, is employed to investigate the performance of PM6:Y6:NPs PSCs. First, our modeling results have been fitted in Fig. 9 with the experimental *J–V* characteristics of reference PM6:Y6 PSC, reported by Li group^165^. In the electrical modeling, we set *WF*_*C*_ = 3.72 eV^165^, which is the Ag WF modified by PDINN. Electron and hole mobilities of PM6:Y6 active layer with the weight ratio of 1:1.2 and thickness of 150 nm, determined by the space charge limited current method and reported in ref.^164^, are 5.90 × 10^–4^ cm^2^ V^−1^ s^−1^ and 2.00 × 10^–4^ cm^2^ V^−1^ s^−1^, respectively. Second, through the HM calculation of the absorption spectrum of PM6:Y6 film embedding Ag, Au, Al, and Cu NPs along with the size optimization of the NPs, the most appropriate metal with optimized size for incorporating into PM6:Y6 blend is found. Third, the *J–V* characteristics of PM6:Y6:NPs PSC with the optimized conditions of NPs, which is Ag NPs with mean size of 20 nm ranged from 5 to 35 nm, is calculated with our semi-analytical modeling and shown in Fig. 9. From this figure and the photovoltaic parameters summarized in Table 3, it is found that the PM6:Y6:NPs PSC demonstrates a high PCE of 18.86%, improved by 10.9% compared to the PM6:Y6 PSC, due to the improved *J*_*sc*_ and FF.

**Table 3:** Photovoltaic parameters of the PM6:Y6 PSC and PM6:Y6:NPs PSC.

	*J*_*sc*_ (mA/cm^2^)	*V*_*oc*_ (V)	FF (%)	PCE (%)
Experimental-PM6:Y6 PSC^165^	25.89	0.847	78.59	17.23
Modeling-PM6:Y6 PSC	25.73	0.847	78.02	17.00
Modeling-PM6:Y6:NPs PSC	27.74	0.849	80.07	18.86

**Figure 10:**
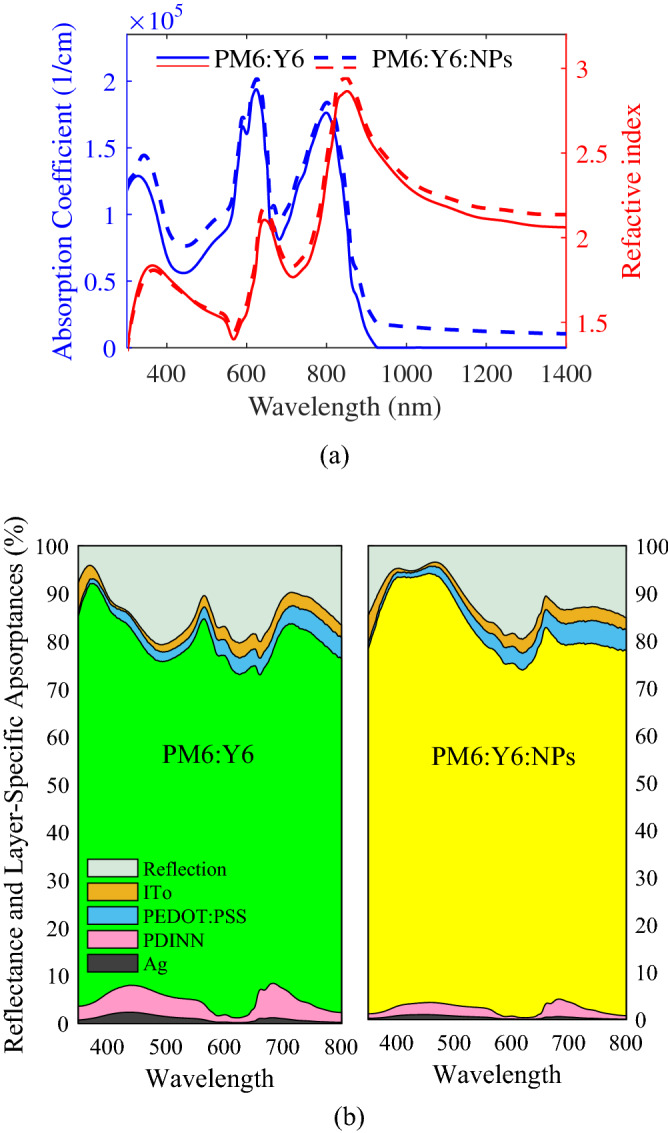
(**a**) Absorption coefficient (left axis) and refractive index (right axis) of PM6:Y6 blend derived by ellipsometry of extinction coefficient given in Ref.^167^, and of PM6:Y6:NPs calculated by the HT. (**b**) The percentage of absorbed photons in each layer and reflected photons from the PM6:Y6 PSC (left) and PM6:Y6:NPs PSC (right).

Enhanced *J*_*sc*_ confirms the improvement of light harvesting in the active layer of PM6:Y6:NPs PSC. Hence, the positive effects of adding Ag NPs to the PM6:Y6:NPs PSC, which are stronger absorption coefficient (see left axis of Fig. 10a) and the increased percentage of absorbed photons in the active layer (see Fig. 10b), prevail the negative influence, which is increased trap-assisted recombination due to increase in trapping states emanating from the embedded Ag NPs.

The increased FF of PM6:Y6:NPs PSC most likely arises from the improved conductivity of the PM6:Y6:NPs compared to the PM6:Y6, as can be found from Eq. (16) and Fig. 10a, leading to the better electron transport within the active layer in the presence of Ag NPs^114,166^.

## Conclusion

In conclusion, a semi-analytical optoelectronic modeling that could predict the performance of plasmonic BHJ PSCs where spherical NPs were incorporated into the active layer was demonstrated. Firstly, the effect of incorporation of NPs into the active layer on the optical properties was analytically modeled by the homogenization theory, considering a disordered array of NPs with size dispersion. Secondly, the percentage of useful absorption by the active layer was calculated by transfer matrix method, in which the number of photons related to absorption in the non-active layers, interference induced by electrodes, and scattered light escaping from the PSC in all directions were subtracted from the total absorbed photons in the PSC. Finally, *J–V* characteristics of plasmonic PSCs were modeled by coupled Poisson, continuity, and drift–diffusion equations. Then, by comparing the results obtained by the semi-analytical modeling with the experimental data reported in the literature for the photovoltaic parameters of P3HT:PCBM:NPs PSCs, which showed a good agreement, our modeling approach was verified. Therefore, for realistic prediction of the photovoltaic parameters of a new high efficiency plasmonic PM6:Y6 PSC, the modeling was applied and yielded that incorporating Ag NPs into PM6:Y6 active layer led to 10.9% improvement in the PCE, from 17 to 18.86%.

## Data Availability

The source code of this work will be made available from the corresponding author upon reasonable request.
